# Living with floating vegetation invasions

**DOI:** 10.1007/s13280-020-01360-6

**Published:** 2020-07-28

**Authors:** Fritz Kleinschroth, R. Scott Winton, Elisa Calamita, Fabian Niggemann, Martina Botter, Bernhard Wehrli, Jaboury Ghazoul

**Affiliations:** 1 Ecosystem Management, Institute of Terrestrial Ecosystems, Department of Environmental Systems Science, ETH Zurich, Universitätsstr. 16, 8092 Zurich, Switzerland; 2 Institute of Biogeochemistry and Pollutant Dynamics, Department of Environmental Systems Science, ETH Zurich, Universitätsstr. 16, 8092 Zurich, Switzerland; 3 Surface Waters – Research and Management, Eawag, Swiss Federal Institute of Aquatic Science and Technology, 6047 Kastanienbaum, Switzerland; 4 VISTA Remote Sensing in Geosciences GmbH, Gabelsbergerstr. 51, 80333 Munich, Germany; 5 Institute of Environmental Engineering, ETH Zurich, Stefano-Franscini-Platz 3, 8093 Zurich, Switzerland; 6 Prince Bernhard Chair of International Nature Conservation, Ecology and Biodiversity, Department of Biology, Utrecht University, Utrecht, The Netherlands; 7 Centre for Sustainable Forests and Landscapes, University of Edinburgh, Edinburgh, EH9 3JT Scotland

**Keywords:** Biological invasions, Dams, Google earth engine, Land cover change, Urbanization, Water-energy-food nexus

## Abstract

Invasions of water bodies by floating vegetation, including water hyacinth (*Eichhornia crassipes*), are a huge global problem for fisheries, hydropower generation, and transportation. We analyzed floating plant coverage on 20 reservoirs across the world’s tropics and subtropics, using > 30 year time-series of LANDSAT remote-sensing imagery. Despite decades of costly weed control, floating invasion severity is increasing. Floating plant coverage correlates with expanding urban land cover in catchments, implicating urban nutrient sources as plausible drivers. Floating vegetation invasions have undeniable societal costs, but also provide benefits. Water hyacinths efficiently absorb nutrients from eutrophic waters, mitigating nutrient pollution problems. When washed up on shores, plants may become compost, increasing soil fertility. The biomass is increasingly used as a renewable biofuel. We propose a more nuanced perspective on these invasions moving away from futile eradication attempts towards an ecosystem management strategy that minimizes negative impacts while integrating potential social and environmental benefits.

**Electronic supplementary material:**

The online version of this article (10.1007/s13280-020-01360-6) contains supplementary material, which is available to authorized users.

## Introduction

More than 50 years ago, *Science* published a landmark article, “Aquatic Weeds” (Holm et al. 1969). The authors reviewed a global assortment of floating vegetation invasions that incurred substantial costs by blocking boat traffic, degrading the capacity to irrigate crops, and interfering with hydropower generation. The authors further suggested that such invasions are “the symptoms of our failure to manage our resources.” They argued that the management remedy to this global crisis is the stepping up of biological quarantines and physical, chemical and/or biological control interventions.

Since Holm et al. (1969) sounded the alarm, research on aquatic weed invasions has accelerated, while very substantial resources have been allocated to control measures (Villamagna and Murphy 2010; Hussner et al. 2017). For example, the Spanish government spent 20.9 million US$ over 4 years to use heavy machinery to physically remove floating vegetation along a 75 km stretch of the Guadiana river (EPPO 2009); two million US$ were spent on personnel costs to run a 3-year project on biological control of water hyacinths in Benin (De Groote et al. 2003); and a single herbicide spraying campaign on Hartebeespoort dam in South Africa cost an equivalent of 200’000 US$ (Van Wyk and Van Wilgen 2002). Although local examples of successful control exist (Wainger et al. 2018), aquatic weed invasions continue to persist in tropical and subtropical river systems to this day, and are even expanding to higher latitudes with warming climates (Kriticos and Brunel 2016).

To assess trends and future development of floating vegetation we need to understand patterns of floating vegetation invasions in space and time, and relate their distribution to potential drivers such as sources of anthropogenic pollution in the catchment. We expect that, despite control efforts, floating vegetation invasions will persist in the warm regions of the world as long as the nutrient inputs that drive them continue to worsen. If there is no prospect of eradication, then we need to learn how to live with such invasions by applying an ecosystem management approach that acknowledges new ecosystem realities, and seeks to manage invasive floating vegetation as an integral element of the system. In doing so, there is the potential to reduce the scale and costs of control measures, and even to harness potential benefits of floating vegetation.

Reservoirs are particularly useful for studying floating vegetation at a global scale because they act as choke points, where accumulated floating plant masses can be detected from space (Coetzee et al. 2017). Reservoirs are particularly vulnerable to water hyacinth (*Eichhornia crassipes*), considered one of the world’s worst invasive weeds (Holm et al. 1977; Nentwig et al. 2018). To assess dynamics of water hyacinth and other floating species cover over time, we analyze three decades of floating plant cover for 20 reservoirs around the world in which water hyacinth invasions have been documented. We track catchment land cover changes over the same time period to assess potential changes in nutrient sources (i.e. agriculture, urbanization). Finally, we convert water hyacinth coverage into biomass and nutrient content (phosphorus, nitrogen) using synthetic mean values from literature to assess the plants’ potential to mitigate aquatic nutrient pollution and serve as a source of biofuel and fertilizer.

## Materials and Methods

### Study sites

**Fig. 1:**
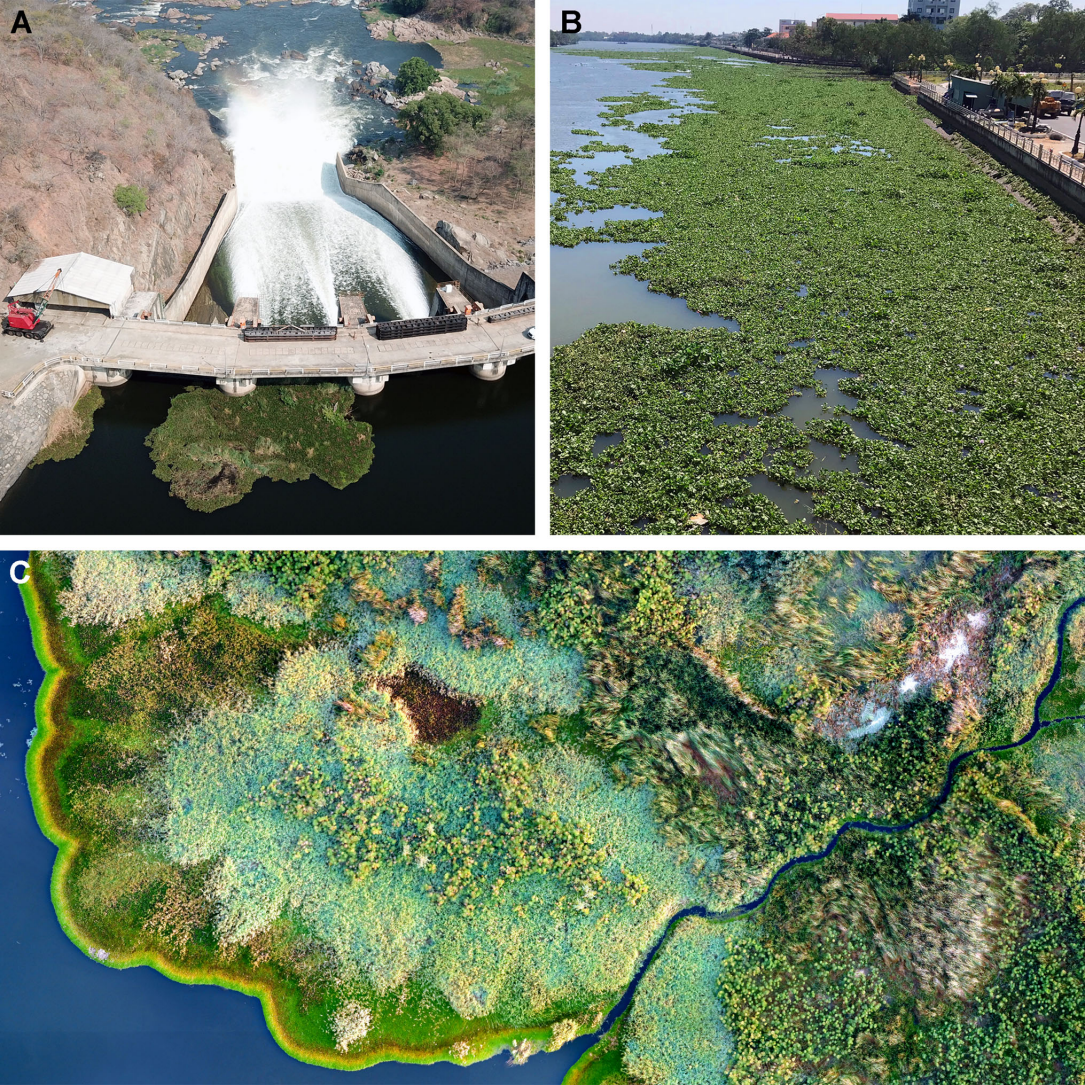
**A** Kafue gorge dam in Zambia with floating vegetation trapped at the spillway (picture by ATEC-3D). **B** Carpets of floating water hyacinths on a tributary of the Vam Co Dong River in Vietnam. **C** Part of the Kafue flats wetland in Zambia. The bright green fringe along the shore is composed of two exotic floating plant species, water hyacinth and Amazon frogbit (picture by ATEC-3D)

Dams are the places in river systems that trap floating vegetation and provide stagnant waters where they accumulate (Fig. 1a). A literature search on 19 June 2019 in Web of Science, using the search terms ((“water hyacinth” OR “Eichhornia crassipes”) AND (reservoir OR dam)) generated 132 hits. Based on abstract and full text search, we selected all studies that reported mass occurrences of *E. crassipes* in dammed reservoirs and regulated river systems. Together with literature that we found through snowballing and a search with the same keywords in Google Scholar to account for grey literature, we found 65 studies fulfilling these criteria (Table S1). We sorted the list by continent and randomly selected 20 sites ensuring equal geographic representation across the Americas, Africa, Asia and Europe (Table 1). This allowed us to study the temporal and spatial dynamics of floating vegetation invasions on globally distributed sample reservoirs.

**Table 1:** Overview of the study sites and floating vegetation cover summary (for further information see Table S1)

#	Name	Country	Catchment area (km^2^)	Water extent (km^2^)^a^	Mean vegetation cover (km^2^)^b^	Mean vegetation cover (%)^b^	Max. vegetation cover (km^2^)^c^	Max. vegetation cover (%)^c^
1.	Bailianhe	China	1733	20.56	0.49	2.4	5.80	28.21
2.	Batujai	Indonesia	184	5.60	0.31	5.53	2.75	49.09
3.	Bellandur	India	149	1.85	0.3	16.18	1.77	95.91
4.	Chao Phraya	Thailand	117 900	6.57	0.26	3.99	3.67	55.83
5.	Chivero	Zimbabwe	2247	20.79	0.94	4.54	7.51	36.10
6.	Guadiana	Spain	48 420	23.01	0.55	2.4	4.04	17.57
7.	Hartebeespoort	South Africa	4028	17.15	1.78	10.36	9.04	52.69
8.	Kafue gorge	Zambia	152 800	10.66	1.17	9.99	5.49	51.48
9.	Kanewal	India	216	3.11	0.84	26.87	3.09	99.28
10.	Koka	Ethiopia	11 140	130.08	1.49	1.15	18.24	14.02
11.	Mariquita	Mexico	176	3.38	0.71	21.0	2.49	73.81
12.	Minjiang	China	52450	52.59	2.26	4.3	23.28	44.26
13.	Petrobras	Brazil	200	1.78	0.35	19.72	1.78	99.75
14.	Ross Barnet	USA	7680	87.99	0.49	0.55	3.06	3.47
15.	Salto Grande	Brazil	38 740	5.57	0.2	3.59	1.16	20.75
16.	Taabo	Ivory Coast	59 610	25.21	0.5	0.2	8.65	34.30
17.	Tapacurá	Brazil	476	5.33	0.29	5.5	1.86	34.91
18.	Tomine	Colombia	356	20.91	0.18	0.85	2.71	12.95
19.	Valsequillo	Mexico	4033	23.38	5.14	21.97	16.39	70.08
20.	Vam Co Dong	Vietnam	6452	5.35	0.3	5.53	2.89	54.05

^a^Based on surface water occurrence threshold (see supplemental information) (Pekel et al. 2016)

^b^Based on observed floating vegetation cover in any given 2-month interval (Fig. 2)

^c^Highest floating vegetation cover detected in any 2-month interval during the full study period

### Floating vegetation detection

Floating vegetation cover on reservoirs can be detected through optical remote sensing due to the clear difference in spectral reflectance to open water and to submerged vegetation and algae that occur inside the water column (Albright et al. 2004; Khanna et al. 2012; Thamaga and Dube 2018). For the selected reservoirs, we produced time series of floating vegetation cover. This approach has been possible through the availability of cloud-based access to the entire Landsat archive from 1984 to 2018, provided by Google Earth Engine. The possibility to run individually developed algorithms on this huge archive makes it possible to evaluate the long time series and distributed areas presented in this paper. For the observed time-span, we masked the water surface area and extracted the part of it that was covered with floating vegetation in each of 2-month intervals that had imagery available. The grouping into 2-month time steps helped to reduce errors due to missing data from cloud cover and unavailable imagery. Bi-monthly averaging also reduced the error introduced through individual days where wind and currents spread the floating plants over particularly large areas with low density coverage. Especially before 1999, data gaps occur due to missing imagery. For Bellandur, Hartebeespoort and Minjiang, time series were cropped to 2000, 1990 and 1995 respectively, as dams were only completed by these years. While we targeted reservoirs with the presence of water hyacinths, carpets of floating vegetation often also include other species (Hestir et al. 2008; Cavalli et al. 2009). Our long-term automated detection method was not able to differentiate different species of floating plants, but we argue that grouping them makes sense, given that the main problems they cause (such as physical obstruction of waterways) are independent of the species.

We defined water bodies based on the global surface water occurrence data (Pekel et al. 2016). To account for seasonal variations in the water surface, we applied thresholds between 5 and 75% occurrence based on case-by-case visual assessment of Google Earth high resolution imagery for multiple years (see Table S1). In a next step, the Normalized Difference Vegetation Index (NDVI) was calculated using Landsat data to identify vegetation based on its spectral reflectance in the red and near infrared spectrum. The NDVI uses the fact that plants strongly absorb visible light (400–700 nm) and reflect near-infrared light (700–1100 nm), resulting in an index from + 1 to − 1 describing the vitality of vegetation. The fact that water poorly reflects infrared light facilitates the differentiation between vegetation and water (Robles et al. 2015). A 2-month composite of satellite data was used to minimize the influence of data gaps due to clouds and cloud shadows. Using a NDVI threshold of 0.3 and masking all areas outside the water body results in a layer of floating vegetation.

To analyze the long-term trend in floating vegetation invasions, we normalized the area covered by floating plants for each site between 0 and 1. We then grouped these values across sites in 5-year intervals. We used a pairwise Wilcoxon rank sum test with a Benjamini & Hochberg adjustment for *p*-values (Benjamini and Hochberg 1995) to compare each of the seven intervals with each other. *P* values smaller than 0.05 were considered significant.

Since we excluded seasonally flooded areas, the automated approach to classify floating vegetation is not appropriate to detect overlaps between seasonally dry areas and those covered by floating vegetation. For two reservoirs with strong seasonal water level alterations, we therefore, manually digitized water surface area and floating vegetation during one hydrologic season (2017/2018) to identify areas dominated by floating vegetation that run dry occasionally. Comparison of floating vegetation on permanent water bodies and seasonally dry areas, gave an indication where plants strand, senesce and decompose on land. Both reservoirs, Lake Koka in Ethiopia and Lake Batujai in Indonesia, showed signs of agricultural use of the temporarily inundated areas, detected from personal field observations (Koka) and from Google Earth imagery (Batujai).

### Land cover modelling

We extracted land cover change from the ESA CCI land cover time series from 1992 to 2015 (Li et al. 2017) within catchment polygons at Pfafstetter levels 5–10 from the HydroSheds database (Lehner and Grill 2013). We then used linear regression models to correlate peak floating vegetation cover with the change in urban and agricultural land-cover within each catchment between 1992 and 2015. As an additional variable we calculated the size of the reservoirs based on the same surface water extent (Pekel et al. 2016) used for floating vegetation detection as described above. The model is limited and could be improved in the future by including other variables such as density of urban areas, availability of wastewater treatment infrastructures and run-off vs. infiltration depending on vegetation and soils.

We used overall peaks in floating vegetation cover to account for the full variability during the study period, given that coverage is close to 0 for all reservoirs at some point in time. We log-transformed values to approximately conform to normality. We used absolute rather than relative values of peak floating vegetation cover. Here, our assumption was that there is a direct link between nutrient inputs from a given land cover area and the amount of vegetation that grows. Only small reservoirs up to 5 km^2^ in size showed up to 100% coverage of floating vegetation, thus limiting the results. For larger reservoirs the overall size was less influential.

### Climate data

We used rainfall data from the CHIRPS dataset (Funk et al. 2014) and temperature data from station data of the Global Historical Climatology Network (Lawrimore et al. 2011). For the full observation period, we calculated bi-monthly annual peak occurrence of rainfall, temperature and floating vegetation. We calculated the time lag (in months) between the occurrence of floating vegetation peaks relative to temperature and rainfall and ordered observations depending on latitudes.

### Nutrient contents in river systems and bound in biomass

For converting areal coverage of water hyacinth to nutrient mass, we use the synthetic mean values of 2.01 (± 0.21 SE) kg dry mass m^−2^ of water hyacinth and 2.18 (± 0.35) % nitrogen and 1.05 (± 0.33) % phosphorus taken from available studies (see Tables S2 and S3). To estimate river nutrient flux we multiplied discharge by concentration. We used discharge values from the Global Runoff Database Centre (GRDC) or from literature. Discharge data were not available for Tapacura reservoir, so we estimated it by generating a catchment area: discharge curve using nearby GRDC stations (Fig. S3). We used the long-term mean of total phosphorus and total nitrogen concentrations reported by the International Centre for Water Resources and Global Change GEMStat water quality database (https://gemstat.org/) (Table S4). We calculated the uncertain fraction of nutrient content in biomass by multiplying the relative standard errors (10.6% for biomass, 16% for N and 31% for P).

All calculations were done using R (R Core Team 2018) with the packages “raster”, “rgdal”, ”rgeos”, ”rnoaa”, ”ggplot2”.

## Results and discussion

### Increasing dominance of floating vegetation

**Fig. 2:**
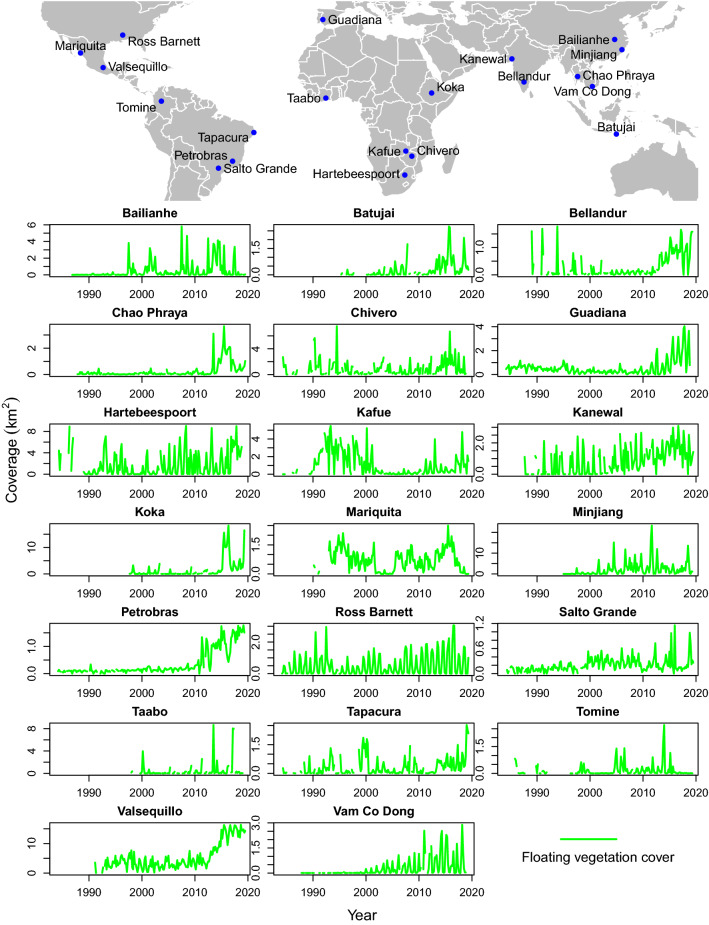
Time series of floating vegetation cover in 20 reservoirs with reported occurrence of water hyacinths since 1984, when frequent, reliable remote sensing data first becomes available. Time series for Minjiang start later, as the dam was built in the 1990s. Other empty values result from data gaps

**Fig. 3:**
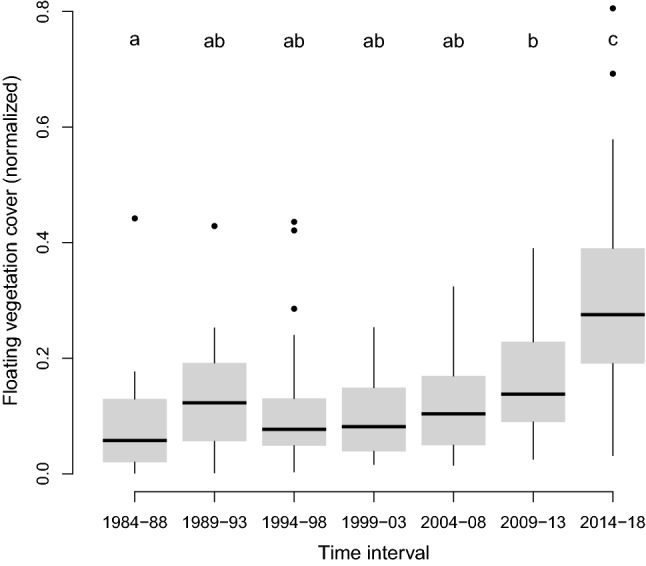
Boxplots of normalized floating vegetation cover across 20 reservoirs between 1984 and 2018 grouped in 5-year intervals. Values for each site are normalized between 0 and 1. Small letters indicate significant differences (*p* < 0.05) between intervals, determined through a pairwise Wilcoxon rank sum test

Our remote sensing analysis of plant coverage on 20 reservoirs indicates that floating vegetation invasions are getting worse (Fig. 2). After fluctuations in the 1980s and 1990s, overall coverage reached significantly higher levels since 2009 (Fig. 3). The one exception is the Kafue Gorge Reservoir in Zambia, which experienced its most intense coverage in 1990s. Even here, following a decade of low plant coverage achieved by intensive nutrient pollution controls and control campaigns from 1998 to 2000 (Chola 2001), floating vegetation has begun to increase again since 2011. For other reservoirs, short-lived low values are explained by specific control campaigns, as for Mariquita in Mexico (Aguilar et al. 2003) and Ross Barnett reservoir in the USA (FTN Associates 2011).

Nearly all sites exhibited seasonal oscillations in floating plant coverage (Fig. 2), as previously documented for Lake Victoria (Albright et al. 2004). In some cases, such as Ross Barnett Reservoir in Mississippi, USA, and Vam Co Dong in Vietnam (Fig. 1 B), the vegetation disappears completely during the cool/dry season. Floating vegetation peaks tend to correlate with annual peaks in temperature and rainfall. Cooler temperatures limit the plants’ growth rate, especially in subtropical or temperate settings. Rainfall delivers nutrients into aquatic ecosystems, facilitating growth, and flushes plant mats out of backwaters into reservoirs (Fig. S1).

### Urban nutrient pollution a likely driver

Continued increases in floating vegetation cover in reservoirs is troubling in view of the resources deployed to fight invasions and the global boom of tropical dam construction that is likely exacerbating the problem (Zarfl et al. 2014). Floating vegetation growth is controlled by the availability of phosphorus and nitrogen (Wilson et al. 2005; Coetzee and Hill 2012; You et al. 2014), and increased nutrient loading linked to changes in land cover could enhance water hyacinth growth. One potential landscape source is expansion of intensive agriculture and associated fertilizer leaching, though we do not find a significant correlation between floating vegetation cover and change in cropland extent in the catchment (linear model, *p* = 0.191). It is possible that agricultural fertilizers are important nutrient sources in some locations, but the best available land cover time series (ESA CCI) does not discriminate between degrees of agricultural intensification, limiting our capacity to thoroughly assess this potential driver.

**Fig. 4:**
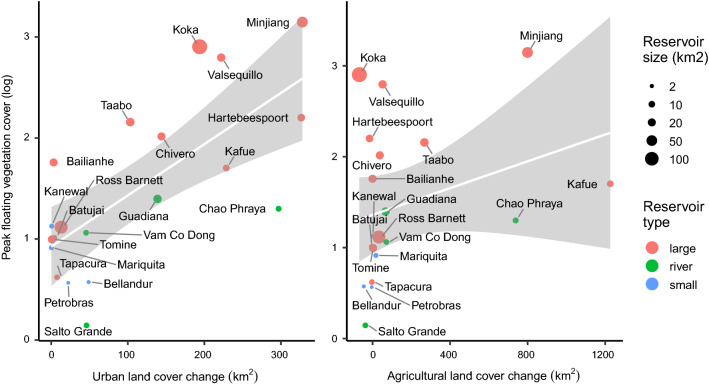
Scatterplot showing the correlation between log-transformed peak floating vegetation cover in reservoirs and change in urban and agricultural land cover from 1992 to 2015 (ESA CCI land cover time series) in each catchment. Scaling of points according to reservoir size, color coding by reservoir type. Regression lines for the full dataset with 95% confidence intervals in gray

An alternative nutrient source could stem from urban land cover and associated municipal wastewater or industrial effluents. We find a strong positive correlation between peak floating vegetation cover and increasing urban land cover (Fig. 4; *p* < 0.001, *R*^2^ = 0.43). Additionally, floating vegetation dominance correlates with reservoir size (*p* = 0.011). Small reservoirs can reach peak floating vegetation coverage > 80%, meaning the size of the water body limits further expansion (Table 1). To account for this limitation, we classified reservoirs < 5 km^2^ as “small”-type. To account for differences in streamflow, we further added the category “river” for run-of-the-river reservoirs with retention times < 2 days. The remaining 12 reservoirs are considered “large”-types that also show a strong correlation with urban land cover change (Fig. 4, *p* = 0.002, *R*^2^ = 0.61).

Despite the unconstrained variability in wastewater treatment across catchments, increasing urban land cover explains up to 61% of variability in floating vegetation coverage. Industrial point-sources are likely to be important nutrient sources for water hyacinth in some places (Sinkala et al. 2002) and they are typically embedded within urbanized areas. In addition, inadequate urban wastewater treatment, a widespread problem in tropical countries that dominate our data set, is likely to be a significant nutrient source driving long-term patterns in the coverage we observe at reservoirs globally. We conclude that a major driver of increasing floating vegetation on reservoirs is nutrient pollution delivered to aquatic ecosystems from urban areas. Given the high rates of urban expansion around the world (Seto et al. 2012) and an exacerbation of nutrient pollution globally (Damania et al. 2019), it is likely that the increasing trend of floating vegetation invasions will continue.

Given that the floating invasion issue is global and will likely persist and worsen alongside urbanization, we propose a more nuanced perspective to management. Because human nutrient inputs drive floating plant growth, dense mats commonly form at sheltered and shallow sites near urban areas (Fig. S2). These places coincide with living and working areas, hence the disruption to human activity. Rapid growth of water hyacinth might be better interpreted as an indicator and symptom of a more serious issue of poor wastewater management rather than a specific problem to be addressed in isolation. Water hyacinth outbreaks also occur in South America, its native range, in polluted surface waters. Invasion is, therefore, not necessarily a function of being an exotic species in a novel environment. Moreover, as long as nutrient loads remain modest, water hyacinth appears to lack the capacity to outcompete co-occurring floating vegetation species (Khanna et al. 2012). Based on drone images, we have found that in regions of the Kafue river system in Zambia, water hyacinths co-exist in association with diverse communities of other (native and non-native) aquatic plant species (Fig. 1c).

### Invasions may mitigate nutrient pollution

Blooms of floating aquatic vegetation are a symptom of nutrient pollution but could become part of solution strategies. Only a reduction of nutrient emissions through, for example, treatment of wastewater and urban runoff, will reduce the underlying cause of aquatic weed invasions. Nonetheless, floating vegetation itself can ameliorate some of the problems of high nutrient loads by its capacity to extract nutrients directly from the water column (Brix 1997; Pilon-Smits 2005). Floating vegetation is regularly used in this way in constructed wetlands to treat wastewater (Reddy and Sutton 1984; Dhote and Dixit 2009) with the proven ability to remove heavy metals from contaminated water bodies through phytoremediation (Jones et al. 2018; Rodríguez-Espinosa et al. 2018). In the absence of effective wastewater treatment facilities, uncontrolled floating vegetation invasions may partly take over the role of mitigating anthropogenic pollution (Rezania et al. 2015). In shallow lake systems, such effects might contribute to the resilience of the lake system, and reduce the likelihood of a transformative system shift to a eutrophic stable state.

**Fig. 5:**
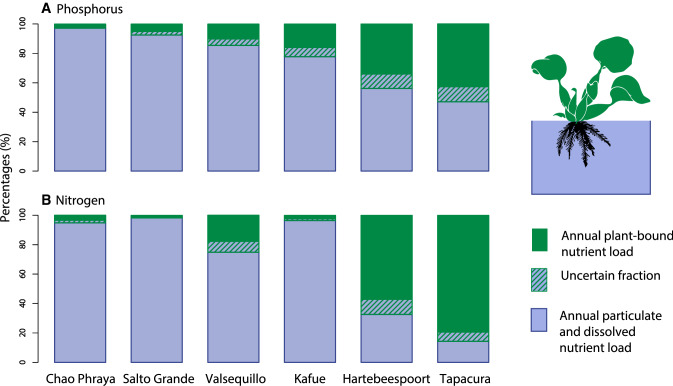
Percentage of total riverine nutrient flux bound to floating vegetation for sub-set of study sites with available total nutrient concentration and discharge data

We evaluated the potential for floating vegetation to absorb nutrients, and found that carpets of floating vegetation on reservoirs represent a major nutrient pool. Based on the area and corresponding biomass detected on our data set of 20 reservoirs, the annual peak phosphorus content of these plants is 1.1 Gg, approximately 3% of the increase in phosphorus fertilizer demand for Africa south of the Sahara from 2014 to 2018 (FAO 2015). An examination of specific catchments reveals that nutrients bound to floating plants represent a substantial component of local riverine nutrient fluxes. This is most pronounced in smaller catchments with a high degree of urbanization, such as Hartebeespoort in South Africa (39% P, 61% N) and Tapacurá in Brazil (48% P, 82% N). Water hyacinth may also be important in more oligotrophic large catchments, such as the Kafue in Zambia where 19% and 3% of annual P and N fluxes are bound within floating plants (Fig. 5). The proportion is more modest in smaller, run-of-the-river reservoirs with a large catchment area, such as Chao Phraya in Thailand.

Floating plants in reservoirs represent a long-term removal of nutrients from surface water when their biomass is removed mechanically, or sequestered through sedimentation which may capture 8% of water hyacinth detritus annually (Reddy and DeBusk 1991). But even in the absence of significant export processes, nutrients bound to floating plants remain relatively unavailable to biota and may serve as an important buffer to prevent aquatic ecosystem collapse. A host of fish and invertebrate herbivores consume water hyacinth, themselves attracting predators such as larger fish and birds (Gopal 1987; Njiru et al. 2002). Thus a complex food web assembles atop a water hyacinth foundation. Without water hyacinth to lock up nutrients in biomass, there is a risk of reservoirs becoming increasingly eutrophic and dominated by phytoplankton or cyanobacteria (Scheffer et al. 1993). Floating vegetation, even if it doesn’t permanently remove nutrients, likely reduces nutrient availabilities in the water, and mitigates the undesirable effects of nutrient pollution to fish populations through algal blooms and hypoxia. Further evidence for its importance as a nutrient buffer are studies documenting rapid increases in dissolved nutrient concentrations following chemical spraying or mechanical shredding (Mangas-Ramírez and Elías-Gutiérrez 2004; Reddy and Sacco 2010). For at least some local managers, the nutrient buffering benefits provided by water hyacinth are already common knowledge (Sinkala et al. 2002).

### From problem to resource

**Fig. 6:**
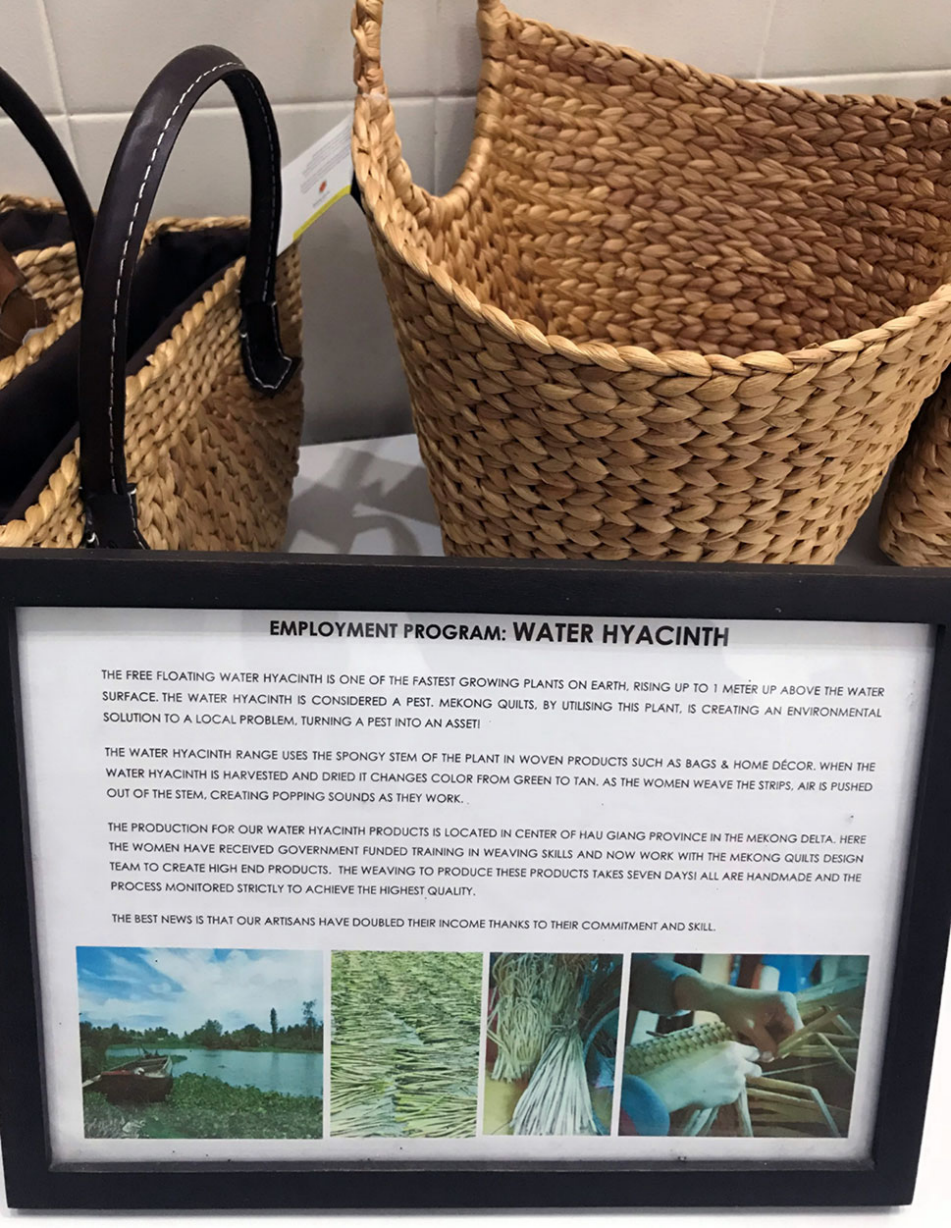
Baskets made of water hyacinths from a community project, for sale in a shop in Phnom Penh, Cambodia

Floating vegetation control programmes are costly (Mara 1976; Epanchin-Niell 2017; Jardine and Sanchirico 2018). They are also likely to be ineffective in the long term unless water nutrient levels are managed. A systems oriented approach to managing aquatic weeds is needed, one that takes account of inputs and outputs across spatial scales and ecosystem boundaries. Such an approach would benefit from recognizing that water hyacinth, while certainly a problem, can also be part of the solution, for example by reducing nutrient levels in watercourses, removing heavy metals (Chunkao et al. 2012), as well as providing new income opportunities (Fig. 6).

**Fig. 7:**
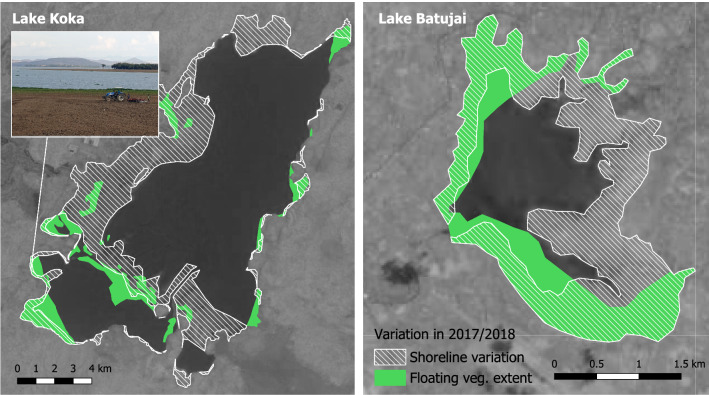
Visual interpretation of shoreline variation and floating vegetation coverage from Landsat and Sentinel 2 images collected between September 2017 and August 2018 from Lake Koka and Lake Batujai sites. Reservoirs in flat topographies show strong season fluctuations in the shoreline (hashed area). The areas that dry out coincide by 50% with those that were (at least temporarily) covered with floating vegetation (green areas). In the overlapping areas, floating vegetation becomes stranded and can serve as compost, as shown in the inset photograph, taken in March 2019

Given their high nutrient contents, stranded floating plants are readily available as green manure (Gunnarsson and Petersen 2007). For seasonally flooded areas of Lake Koka in Ethiopia and Lake Batujai in Indonesia we show how stranded floating plants transport nutrients out of the water back to the land where they fertilize croplands (Fig. 7). In 2018, 48% or 7.34 km^2^ (Koka) and 77% or 2.1 km^2^ (Batujai) of floating vegetation cover was stranded on the shore and at least partially ploughed into agricultural fields. The sequestration of nutrients from the water column, and their subsequent transport to land systems during floods and flood recessions, can deliver the dual benefit of reducing eutrophication risk while replenishing soil fertility. By substituting water hyacinth for synthetic fertilizers, farmers avoid the cost of the latter, as well as the risk of further nutrient run-off into watercourses.

The caloric value of the plant biomass is increasingly acknowledged as a benefit provided by floating vegetation (Shanab et al. 2018; Wang et al. 2019). Water hyacinth is being utilized for bioethanol and biogas production (Wang and Calderon 2012; Hernández-Shek et al. 2016) and the potential for small scale local energy generation in water hyacinth affected areas is high (Wilkie and Evans 2010) despite the challenge of the plant’s high water content (Coetzee et al. 2017). Examples are projects in Niger (Almoustapha et al. 2009) and Kenya (Grist et al. 2018), where biogas from water hyacinths is successfully used as a substitute for wood fuel. Our set of 20 reservoirs annually generates roughly 220 Gg of floating vegetation biomass (Table 1, Table S2), which at a conversion rate of 0.28 m^3^ biogas per dry kg (Wolverton and Mcdonald 1981), could annually produce 0.13 TWh of electricity (at 2 kWh per m^3^ of biogas), worth roughly 19 million US$ in Kenya where energy costs 15 US cents per kWh (as of march 2019). Dam sites seem particularly useful locations for biofuel plants due to the accumulating of plant material and the available infrastructure for plant collection and access to electricity grids.

## Conclusions

Our analysis provides a global picture of increasing floating vegetation invasions during more than three decades, alongside a strong urbanization trend. As nutrient pollution from urban areas continues to increase, invasive floating vegetation can at least partially fulfill important water purification functions, buffering further negative consequences for aquatic ecosystems and water users. In the context of adaptive ecosystem management (Heinimann 2010; Nanda et al. 2018), efforts to manage floating vegetation invasions should not be focused on unrealistic targets to eliminate invasive species. A more effective long-term strategy would be to work with the seasonal dynamics of the hydrological and biological system to make best use of the purification and fertilization services as well as the additional biomass of the floating plants.

## Electronic supplementary material

Below is the link to the electronic supplementary material.

Supplementary material 1 (PDF 1185 kb)

## Data Availability

The data generated and analysed during the current study are publicly available with the DOI 10.3929/ethz-b-000420432 under the following link: https://www.research-collection.ethz.ch/handle/20.500.11850/420432bzw.
