# Supplementary material for: Living with floating vegetation invasions
Source: Ambio. 2020 Jul 28;50(1):125–37. doi: 10.1007/s13280-020-01360-6 (PMC7708604; doi:10.1007/s13280-020-01360-6)
Supplement: Supplementary file 1 — Supplementary material 1 (PDF 1185 kb) [file 13280_2020_1360_MOESM1_ESM.pdf]

Ambio

Electronic Supplementary Material
This supplementary material has not been peer reviewed.

Living with floating vegetation invasions

Fritz Kleinschroth*[1], R. Scott Winton*[2,3], Elisa Calamita[2,3], Fabian Niggemann[4], Martina Botter[5], Bernhard Wehrli[2,3], Jaboury Ghazoul[1]

[1] Ecosystem Management, Institute of Terrestrial Ecosystems, Department of Environmental Systems Science, ETH Zurich, Universitätsstr. 16, 8092 Zurich, Switzerland

[2] Institute of Biogeochemistry and Pollutant Dynamics, Department of Environmental Systems Science, ETH Zurich, Universitätsstr. 16, 8092 Zurich, Switzerland

[3]Eawag, Swiss Federal Institute of Aquatic Science and Technology, Surface Waters – Research and Management, 6047 Kastanienbaum, Switzerland

[4]VISTA Remote Sensing in Geosciences GmbH, Gabelsbergerstr. 51, 80333 Munich, Germany

[5] Institute of Environmental Engineering, ETH Zurich, Stefano-Franscini-Platz 3, 8093 Zurich, Switzerland

*These authors contributed equally to this work. Emails: klfritz@ethz.ch, scott.winton@gmail.com

Contents

Supplementary Methods 2
Supplementary Figures 3
Supplementary Tables 6
References 11

Supplementary Methods

Kafue River sampling and nutrient loading calculations

We sampled surface water from the Kafue River at the bridge near Kafue town upstream of the Kafue Gorge reservoir four times at roughly equal intervals from March 2018 through February 2019. We analyzed triplicate samples for total phosphorus and total nitrogen on a Skalar flow-injection analyzer following autoclave acid digestion. To calculate annual fluxes we took the mean of each set of triplicates and then the mean concentration from each sampling time point to generate an annual mean concentration for nitrogen and phosphorus. We multiplied the annual mean concentration by annual mean discharge to generate an estimated non-plant annual nutrient load. This estimate is susceptible to error since concentrations and discharge vary seasonally, but it serves simply to provide context for the nutrients bound within floating vegetation and so precision is not critical.

Discharge estimation for Tapacura

We estimated Tapacura discharge using the neighboring GRDC stations (https://www.bafg.de/GRDC). Catchment area is 478.3 km^2 and we estimate discharge to be 2.4 m^3 s^{-1} (Fig. S2)

Supplementary Figures

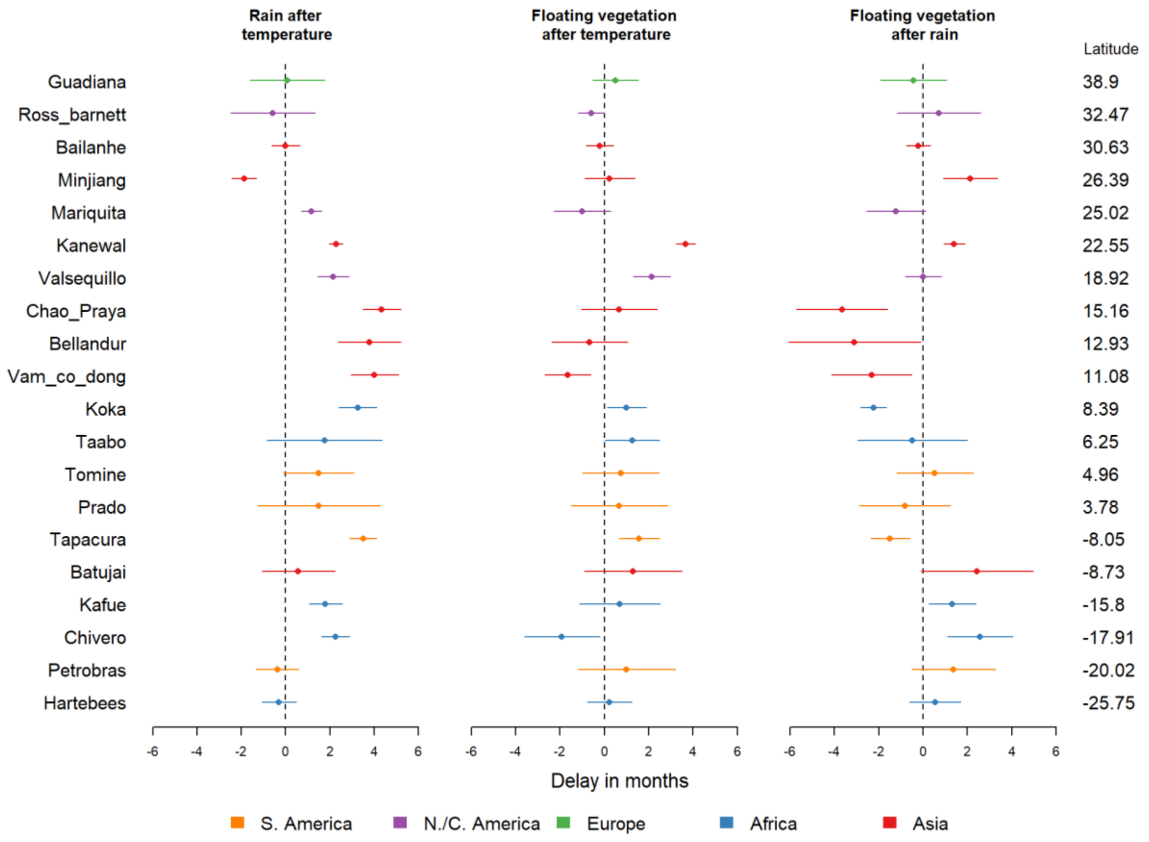

Figure S1: Time lag in months between peak occurrence in temperature, rainfall and floating vegetation coverage. Zero indicates that peaks coincide within the same 2-month interval. Negative values indicate that the order stated in the title was reversed. At +/- 6 months before/after becomes irrelevant. Dots and error bars denote means and 95% confidence intervals. Sorting based on latitude, color groups based on continents.

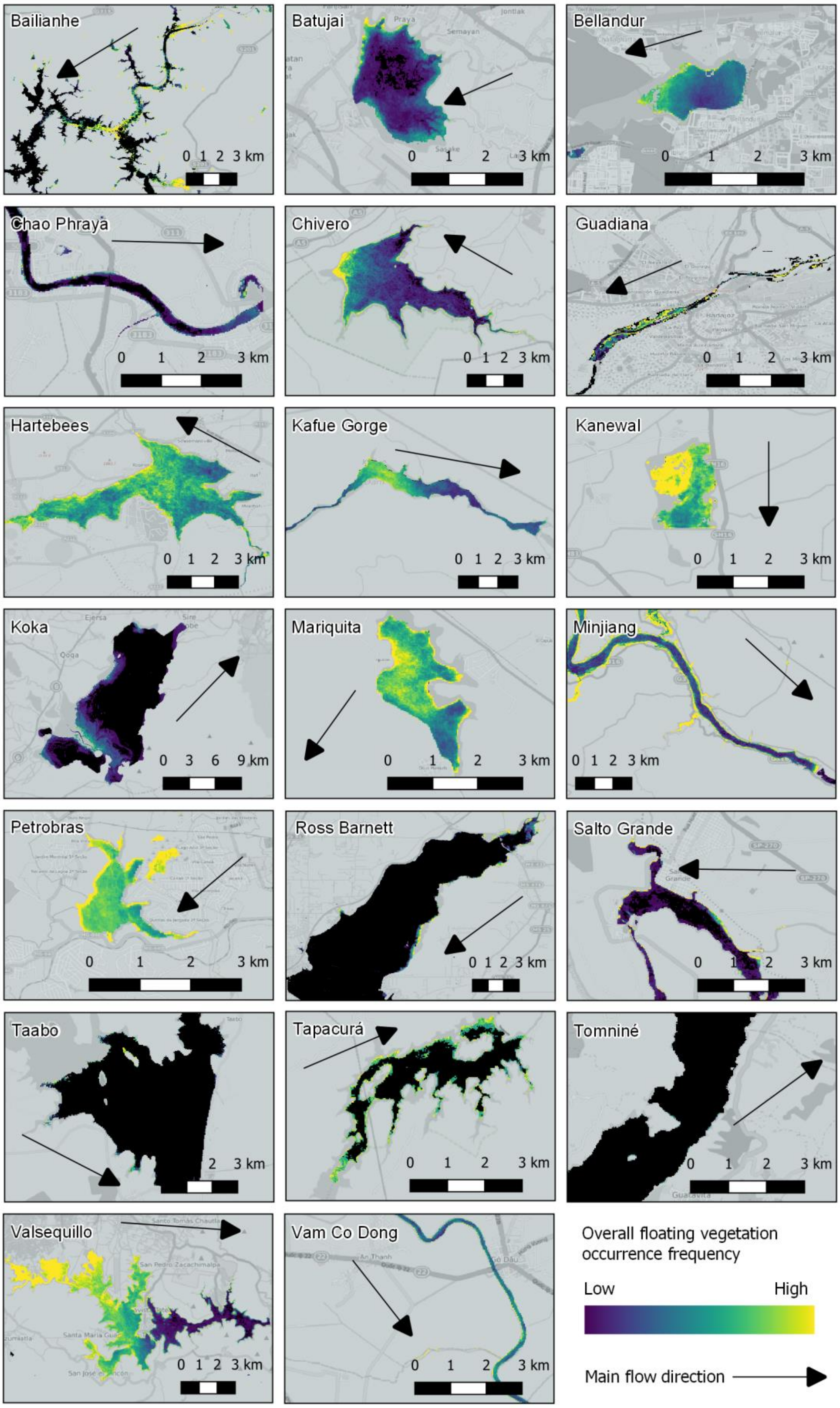

Figure S2: Spatially explicit occurrence frequency of floating vegetation in 20 reservoirs. Background: Open Street Map (www.openstreetmap.org).

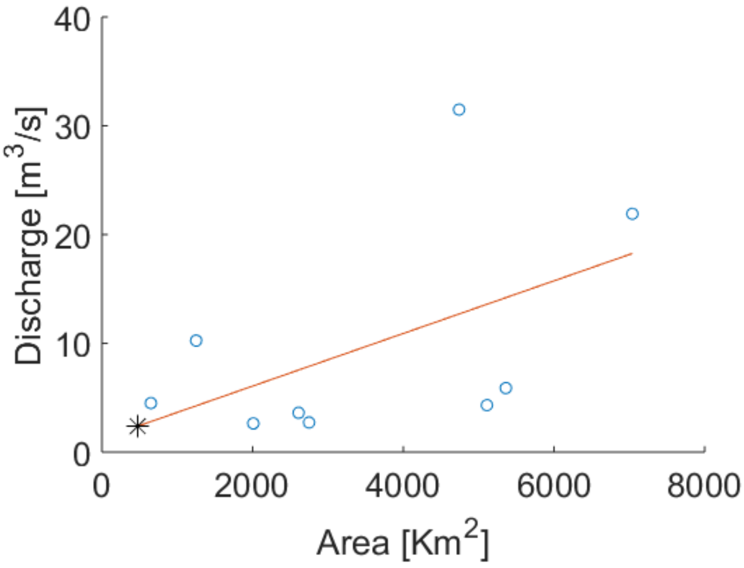

Figure S3. Catchment area-discharge relationship from GRDC stations (https://www.bafg.de/GRDC) near Tapacura reservoir in Brazil.

Supplementary Tables

Table S1: Documented cases of water hyacinth occurrences in reservoirs and river systems and their source in the literature, sorted by continents. Based on searches in Web of Science and Google Scholar (see methods).

	Name	Country	Coordinates	References	selected	Surface water threshold (%)
**South America**						
1.	Aimores	Brazil	19°29'49.86"S, 41° 1'22.91"W	(Pitelli et al. 2014a)		
2.	Cursai	Brazil	7°52'39.00"S, 35°10'34.58"W	(Moura Junior et al. 2019)		
3.	Daule-Peripa	Ecuador	0°53'42.00"S, 79°42'33.00"W	(Nguyen et al. 2015)		
4.	Garcas	Brazil	23°38'47.73"S, 46°37'26.04"W	(Bicudo et al. 2007; Crossetti and Bicudo 2008; Crossetti et al. 2019)		
5.	Guri	Venezuela	7° 5'3.78"N, 62°55'14.64"W	(Vilarrubia and Cova 1993)		
6.	Itaipu	Brazil	22°57'35.58"S, 43° 2'24.52"W	(Bini et al. 1999; Thomaz et al. 2006)		
7.	Lagoa de Petrobras	Brazil	20°1′22.36″ S, 44°6′49.06″W	(Quintao et al. 2013)	X	55
8.	Manamo	Venezuela	8°52'41.94"N, 62° 2'49.27"W	(Olivares and Colonnello 2000)		
9.	Medalha	Brazil	19°34'34.29"S, 57° 0'46.78"W	(Peixoto et al. 2016)		
10.	Muña	Colombia	4°31'48.69"N, 74°15'11.75"W	(Giraldo and Garzón 2002; Villamagna and Murphy 2010)		
11.	Oros	Brazil	6°16'25.15"S, 38°56'10.61"W	(Rocha et al. 2015)		
12.	Ourinhos	Brazil	23° 4'3.71"S, 49°50'10.35"W	(Cunha-Santino et al. 2016)		
13.	Pampulha	Brazil	19°50'55.97"S, 43°58'9.82"W	(Pinto-Coelho and Greco 1999; Villamagna and Murphy 2010)		
14.	Pantanal Lake	Brazil	19° 1'13.13"S, 57°33'0.64"W	(Peixoto et al. 2016)		
15.	Paranoá	Brazil	15°47'24.20"S, 47°48'50.07"W	(Domingues et al. 2017)		
16.	Piraju	Brazil	23°11'16.87"S, 49°23'2.84"W	(Bianchini Jr et al. 2011)		
17.	Porto Primavera	Brazil	22°26'42.27"S, 52°55'7.38"W	(Martins et al. 2009; Pitelli et al. 2014b)		
18.	Prado	Colombia	3°45'24.55"N, 74°53'10.45"W	(Santos et al. 2012)		
19.	Rodo	Uruguay	34°54'47.21"S, 56°10'1.24"W	(Villamagna and Murphy 2010)		
20.	Salto Grande	Brazil	22°54'10.96"S, 49°59'56.37"W	(Martins et al. 2011)	X	55
21.	Tapacurá	Brazil	8° 2'18.70"S, 35° 9'51.14"W	(Moura Junior et al. 2019)	X	55
22.	Tomine	Colombia	5° 1'46.89"N, 73°49'0.18"W	(Rodriguez et al. 2012)	X	55

	Name	Country	Coordinates	References	selected	Surface water threshold (%)
23.	Vigário	Brazil	22°40'15.43"S, 43°52'51.78"W	(Domingues et al. 2017)		
**Central and North America**						
24.	Corpus Christi	USA	28° 2'45.16"N, 97°52'44.87"W	(Everitt and Yang 2007)		
25.	Cruz Pintada	Mexico	18°27'42.14"N, 99° 2'10.17"W	(Martinez Jimenez and Gomez Balandra 2007)		
26.	Endho	Mexico	20° 7'55.87"N, 99°22'9.17"W	(Lopez 1993)		
27.	Jose Antonio Alzate	Mexico	19°26'56.50"N, 99°39'21.21"W	(Rodriguez et al. 1998)		
28.	Mariquita	Mexico	24°55'29.29"N, 107°32'51.56"W	(Aguilar et al. 2003)	X	25
29.	Ross Barnet	USA	32°30'58.43"N, 89°55'50.22"W	(Ervin et al. 2006; Wersal et al. 2006)	X	75
30.	San Joaquin	USA	38° 2'31.92"N, 121°48'6.24"W	(Hestir et al. 2008; Santos et al. 2009; Khanna et al. 2012; Hopper et al. 2017; Lars et al. 2017)		
31.	Valsequillo	Mexico	18°55'12.48"N, 98°11'30.37"W	(Mangas-Ramírez and Elías-Gutiérrez 2004; Villamagna and Murphy 2010; Rodríguez-Espinosa et al. 2018)	X	5
**Asia**						
32.	Anavilundawa	Sri Lanka	7°42'16.20"N, 79°48'42.24"E	(Gunaratne et al. 2009; Gunaratne et al. 2015)		
33.	Bailianhe	China	30°35'35.03"N, 115°26'55.27"E	(Lu et al. 2007)	X	75
34.	Batujai	Indonesia	8°44'0.16"S, 116°16'7.48"E	(Rahim and Soeprobowati 2019)	X	55
35.	Bellandur	India	12°56'3.90"N, 77°39'46.24"E	(Venugopal 2002)	X	25
36.	Caohai	China	24°58'49.88"N, 102°38'26.44"E	(Zhang et al. 2019)		
37.	Chao Phraya	Thailand	15° 9'30.28"N, 100°10'42.89"E	(Thamasara 1989; Chunkao et al. 2012; Jongmeesuk et al. 2014; Viriyavisuthisakul et al. 2018)	X	55
38.	Deepor Beel	India	26°05′–26°11′N, 91°35′–91°43′E	(Bhattacharyya and Kapil 2010)		
39.	Dianchi	China	24°56'58.09"N, 102°39'41.42"E	(Wang et al. 2012)		
40.	Kanewal	India	22°32'50.77"N, 72°37'0.53"E	(Kumar et al. 2008)	X	35
41.	Minjiang	China	26°18'12.79"N, 118°48'41.10"E	(Xiao et al. 2002; Leiming 2006)	X	55
42.	Okhla	India	28°33'13.57"N, 77°18'37.77"E	(Bansal et al. 2015)		
43.	Pant Sagar	India	24°11'57.97"N, 83° 0'20.72"E	(Mishra et al. 2008; Rai 2009)		
44.	Rawa Pening	Indonesia	7°17'4.51"S, 110°25'59.13"E	(Wulandari et al. 2019)		
45.	Selorejo	Indonesia	7°52'23.07"S, 112°21'40.98"E	(Jennerjahn 2013)		

	Name	Country	Coordinates	References	selected	Surface water threshold (%)
46.	Shahpura lake	India	23°12'16.68"N, 77°25'17.03"E	(Villamagna and Murphy 2010)		
47.	Thimmapuram	India	12°27'15.72"N, 78°13'23.15"E	(Attermeyer et al. 2016)		
48.	Three Gorges Dam	China	30°50'5.11"N, 110°59'46.90"E	(Xiong et al. 2018)		
49.	Vam Co Dong	Vietnam	10°58'22.72"N, 106°17'22.36"E	(Sunwoo et al. 2019; Thi et al.)	X	55
50.	Xinglinwan	China	24°36′01″N, 118°03′55″E	(Wei et al. 2011)		
**Africa**						
51.	Aba Samuel	Ethiopia	8°48'37.02"N, 38°42'24.51"E	(Ingwani et al. 2010)		
52.	Chivero	Zimbabwe	17°53'9.99"S, 30°46'15.48"E	(Magadza 2003; Rommens et al. 2003; Marshall 2005; Villamagna and Murphy 2010; Regina 2012; UNEP 2013; Dube et al. 2017; Muchini et al. 2018)	X	55
53.	Delta Park	South Africa	26° 7'29.91"S, 28° 0'42.34"E	(Katembo et al. 2013)		
54.	Farm Dam	South Africa	26° 1'44.40"S, 27°27'30.01"E	(Katembo et al., 2013)		
55.	Hartebees	South Africa	25°43'49.35"S, 27°51'8.40"E	(Edwards and Musil 1975; Ashton et al. 1979; Hill and Olckers 2000; Coetzee et al. 2007)	X	55
56.	Kafue gorge	Zambia	15°46'47.32"S 28°20'14.60"E	(Chola 2001; Sinkala et al. 2002; Alsterhag and Petersson 2004; WWF 2017)	X	55
57.	Koka	Ethiopia	8°28'5.90"N, 39° 9'18.55"E	(Firehun et al. 2013; Kassaye et al. 2016)	X	75
58.	Kyoga	Uganda	1°29'27.45"N, 32°56'29.58"E	(Ogutu-Ohwayo et al. 2013)		
59.	Letaba	South Africa	23°16'57.27"S, 30°24'15.14"E	(Thamaga and Dube 2018)		
60.	New Year's Dam	South Africa	33°18'3.10"S, 26° 6'45.52"E	(Fraser et al. 2016)		
61.	Nokoué	Benin	6°29'28.40"N, 2°26'1.82"E	(Djihouessi et al. 2017)		
62.	Taabo	Ivory Coast	6°13'35.86"N, 5° 5'28.11"W	(Kouamé et al. 2010)	X	55
63.	Tana	Ethiopia	12° 0'38.55"N, 37°18'59.87"E	(Goshu and Aynalem 2017)		
64.	Victoria	Kenya	0°17'3.91"S, 34°31'35.96"E	(Williams and Hecky 2005)		
**Europe**						
65.	Guadiana	Spain	38°57'16.14"N, 6° 7'6.51"W	(Téllez et al. 2008)	X	55

Table S2. Areal water hyacinth (*Eichornia crassipes*) biomass (dry).

Biomass (kg m^{-2})	Reference
2.13	(Boyd and Scarsbrook 1975)
2.07	(Gopal et al. 1978)*
3.46	(Gopal 1987)
2.40	(Knipling et al. 1970)*
2.11	(Lallana 1980)*
2.21	(Lallana 1981)
3.20	(Neiff et al. 1977)*
1.50	(Penfound and Earle 1948)
1.28	(Penfound 1956)
1.40	(Perez del Viso et al. 1968)*
2.31	(Reddy and Tucker 1983)
1.73	(Sabattini et al. 1983)*
0.72	(Sahai and Sinha 1970)
0.63	(Singh and Sahai 1979)*
2.97	(Wooten and Dodd 1976)*
**2.01**	**mean**
0.83	standard deviation
0.21	standard error
10.63	residual standard error (%)

*Values as reported in Gopal (1987). We were unable to access the original texts.

Table S3. Water hyacinth (*Eichornia crassipes*) nutrient content.

N (%)	P (%)	Reference
1.03	0.43	(Abdalla and Abdel Hafeez 1969)
-	0.53	(Abdelhamid and Gabr 1991)
1.43	0.59	(Abou el Fadl et al. 1970)*
-	1.10	(Agrupia 1953)*
2.50	0.42	(Boyd 1969)
2.64	0.43	(Boyd 1970)*
2.39	0.54	(Boyd and Vickers 1971)
2.20	0.44	(Oliveira 1977)*
-	0.50	(Easley and Shirley 1974)
1.18	0.26	(Gunnarsson and Mattsson 1997)
1.83	0.17	(Howard-Williams and Junk 1976)
3.12	0.43	(Musil and Breen 1977)
1.61	0.31	(Parra and Hortenstine 1974)
2.76	0.53	(Poddar et al. 1991)
2.90	0.50	(Polprasert et al. 1980)*
2.90	0.63	(Wolverton and Mcdonald 1978)
5.01	0.38	(Xie et al. 2004)
**2.38**	**0.48**	**mean**
1.00	0.20	standard deviation
0.27	0.05	standard error
11.28	9.94	residual standard error (%)

*Values as reported in Gopal (1987). We were unable to access the original texts.

Table S4. Riverine nutrient loading for five reservoirs with available total nutrient data for a primary inflow. Hartebeespoort and Valsequillo have more than one river inputs and the loading only refers to inputs from the named tributary.

Reservoir (River)	Q	Concentration[f]		Loading[h]	
	m^3 s^{-1}	*TP (µg L^{-1})*	*TN (µg L^{-1})*	*TP (Mg y^{-1})*	*TN (Mg y^{-1})*
Kafue	989[a]	7.3[g]	289[g]	227	9014
Chao Phraya	454[a]	104	281	1489	4023
Salto Grande (Paranaparema)	279[b]	20	475	176	4179
Valsequillo (Atoyac)	16.6[d]	2170	5480	1139	2877
Hartebeespoort (Crocodile)	4.4[c]	970	1826	136	256
Tapacura	2.4[e]	258	243	19	18

[a]Discharge data from global runoff data centre (GRDC) (https://www.bafg.de/GRDC)
[b]Discharge from (Ferrareze et al. 2014)
[c]Average annual discharge from 1980 to 1989 from (Chutter and Rossouw 1991)
[d]Discharge from (Rodríguez-espinosa et al. 2018)
[e]Discharge calculated via catchment-size discharge relationships built from regional gauged rivers
[f]Long-running mean total nutrient concentration data from the International Centre for Water Resources and Global Change GEMStat water quality database (https://gemstat.org/)
[g]Based on data manually collected by the authors of this study
[h]Calculated from load = Q * concentration

Table. S5. Percentage of total riverine nutrient flux bound to floating vegetation for sub-set of study sites with available total nutrient concentration and discharge data.

Reservoir	Catchment Size	Cover[a]	Nutrient content[b]		Relative importance[c]	
	km^2	*km^2*	*P (Mg)*	*N (Mg)*	*% P*	*% N*
Chao Phraya	117931	3.7	36	177	2.3	4.2
Kafue	44466	5.5	53	263	19.0	2.8
Salto Grande	38735	1.2	12	57	6.2	1.4
Valsequillo	4033	16.4	159	784	12.2	21.4
Hartebeespoort	4029	9.0	87	430	39.0	62.7
Tapacura	476.3	1.9	18.0	88.9	48.0	82.9

[a]Maximum floating vegetation coverage from Fig. 1
[b]Based on areal biomass (Table S2) multiplied by N or P content (Table S3)
[c]Percent of total river-born nutrients (floating plant-bound + annual river nutrient load) represented by floating plants. For annual river nutrient load data and calculations see Table S4

References

Abdalla, A. A., and A. T. Abdel Hafeez. 1969. Some aspects of utilization of water hyacinth (*Eichhornia crassipes*). *PANS Pest Articles & News Summaries* 15: 204–207. doi:10.1080/04345546909415116.

Abdelhamid, A. M., and A. A. Gabr. 1991. Evaluation of water hyacinth as a feed for ruminants. *Archiv für Tierernaehrung* 41: 745–756. doi:10.1080/17450399109428519.

Abou el Fadl, M., S. G. Rizk, A. F. Abdel Ghani, M. el Mofty, M. Khadr, S. Shebata, and F. Farag. 1970. Utilization of water-hyacinth as an organic manure with special reference to water borne helminths. *Journal of Microbiology of the United Arab Republic* 3: 27–34.

Agrupia, F. 1953. Value of water hyacinth as silage. *Philippine Agricultural Journal* 37: 50–56.

Aguilar, J. A., O. M. Camarena, T. D. Center, and G. Bojorquez. 2003. Biological control of waterhyacinth in Sinaloa, Mexico with the weevils Neochetina eichhorniae and N-bruchi. *BIOCONTROL* 48: 595–608. doi:10.1023/A:1025707603627.

Alsterhag, E., and L. Petersson. 2004. Nutrient loading in the Kafue River between Mazabuka and Kafue Town, Zambia. *Minor Field Study 108*: 35 pp.

Ashton, P. J., W. E. Scott, D. J. Steyn, and R. J. Wells. 1979. The Chemical Control Programme Against the Water Hyacinth Eichhornia crassipes (Mart.) Solms on Hartbeespoort Dam: Historical and Practical Aspects. *South African Journal of Science* 75: 303–306.

Attermeyer, K., S. Flury, R. Jayakumar, P. Fiener, K. Steger, V. Arya, F. Wilken, R. Van Geldern, et al. 2016. Invasive floating macrophytes reduce greenhouse gas emissions from a small tropical lake. *Scientific Reports* 6. Nature Publishing Group: 1–10. doi:10.1038/srep20424.

Bansal, S., M. Chakraborty, D. Katyal, and J. K. Garg. 2015. Methane flux from a subtropical reservoir located in the floodplains of River Yamuna, India. *Applied Ecology and Environmental Research* 13: 597–613.

Bhattacharyya, K. G., and N. Kapil. 2010. Impact of urbanization on the quality of water in a natural reservoir: a case study with the Deepor Beel in Guwahati city, India. *Water and Environment Journal* 24: 83–96. doi:10.1111/j.1747-6593.2008.00157.x.

Bianchini Jr, I., M. B. Cunha-Santino, and R. S. Panhota. 2011. Oxygen uptake from aquatic macrophyte decomposition from Piraju Reservoir (Piraju, SP, Brazil). *Brazilian Journal of Biology* 71: 27–35. doi:10.1590/S1519-69842011000100006.

Bicudo, D. D. C., B. M. Fonseca, L. M. Bini, L. O. Crossetti, C. E. D. M. Bicudo, and T. Araujo-Jesus. 2007. Undesirable side-effects of water hyacinth control in a shallow tropical reservoir. *Freshwater Biology* 52: 1120–1133. doi:10.1111/j.1365-2427.2007.01738.x.

Bini, L. M., S. M. Thomaz, K. J. Murphy, and A. F. M. Camargo. 1999. Aquatic macrophyte distribution in relation to water and sediment conditions in the Itaipu Reservoir, Brazil. *Hydrobiologia* 415: 147–154. doi:10.1023/A:1003856629837.

Boyd, C. E. 1969. The nutritive value of three species of water weeds. *Economic Botany* 23: 123–127. doi:10.1007/BF02860614.

Boyd, C. E. 1970. Chemical analyses of some aquatic vascular plants. *Archiv fur Hydrobiologie* 67: 78–85.

Boyd, C. E., and E. Scarsbrook. 1975. Influence of nutrient additions and initial density of plants on production of waterhyacinth Eichhornia crassipes. *Aquatic Botany* 1: 253–261. doi:10.1016/0304-3770(75)90026-1.

Boyd, C. E., and D. H. Vickers. 1971. Variation in the elemental content of Eichhornia crassipes. *Hydrobiologia* 38: 409–414. doi:10.1007/BF00036546.

Chola, P. 2001. Management of aquatic weeds on the Kafue river in Zambia. In *27th WEDC Conference: People and Systems for Water, Sanitation and Health*, ed. R. Scott, 381–382. Lusaka, Zambia.

Chunkao, K., C. Nimpee, and K. Duangmal. 2012. The King's initiatives using water hyacinth to remove heavy metals and plant nutrients from wastewater through Bueng Makkasan in Bangkok, Thailand. *Ecological Engineering* 39: 40–52. doi:10.1016/j.ecoleng.2011.09.006.

Chutter, F., and J. Rossouw. 1991. *The management of phosphate concentrations and algae in Hartebeespoort Dam*. *Water Resources Commission Report*. Vol. 289. Pretoria. doi:10.1192/bjp.112.483.211-a.

Coetzee, J. A., M. J. Byrne, and M. P. Hill. 2007. Impact of nutrients and herbivory by Eccritotarsus catarinensis on the biological control of water hyacinth, Eichhornia crassipes. *Aquatic Botany* 86: 179–186. doi:10.1016/j.aquabot.2006.09.020.

Crossetti, L. O., and C. E. de M. Bicudo. 2008. Adaptations in phytoplankton life strategies to imposed change in a shallow urban tropical eutrophic reservoir, Garcas Reservoir, over 8 years. *HYDROBIOLOGIA* 614: 91–105. doi:10.1007/s10750-008-9539-1.

Crossetti, L. O., D. de C. Bicudo, L. M. Bini, R. B. Dala-Corte, C. Ferragut, and C. E. de Mattos Bicudo. 2019. Phytoplankton species interactions and invasion by Ceratium furcoides are influenced by extreme drought and water-hyacinth removal in a shallow tropical reservoir. *Hydrobiologia* 831: 71–85. doi:10.1007/s10750-018-3607-y.

Cunha-Santino, M. B., A. T. Fushita, A. C. Peret, and I. Bianchini-Junior. 2016. Morphometry and retention time as forcing functions to establishment and maintenance of aquatic macrophytes in a tropical reservoir. *Brazilian Journal of Biology* 76: 673–685. doi:10.1590/1519-6984.24214.

Djihouessi, M., G. Gettel, H. van der Kwast, and M. Aina. 2017. *Management of Eutrophication and water hyacinth in Lake Nokoué (Benin)*. doi:10.13140/RG.2.2.35575.68007.

Domingues, F. D., F. L. R. M. Starling, C. C. Nova, B. R. Loureiro, L. C. e Souza, and C. W. C. Branco. 2017. The control of floating macrophytes by grass carp in net cages: experiments in two tropical hydroelectric reservoirs. *Aquaculture Research* 48: 3356–3368. doi:10.1111/are.13163.

Dube, T., O. Mutanga, M. Sibanda, V. Bangamwabo, and C. Shoko. 2017. Evaluating the performance of the newly-launched Landsat 8 sensor in detecting and mapping the spatial configuration of water hyacinth (Eichhornia crassipes) in inland lakes, Zimbabwe. *Physics and Chemistry of the Earth* 100. Elsevier Ltd: 101–111. doi:10.1016/j.pce.2017.02.015.

Easley, J. F., and R. L. Shirley. 1974. Nutients elements for livestock aquatic plants. *Hyacinth Control Journal* 12: 82–85.

Edwards, D., and C. J. Musil. 1975. Eichhornia crassipes in south africa - A general review. *Journal of the Limnological Society of Southern Africa* 1: 23–27. doi:10.1080/03779688.1975.9632904.

Ervin, G., M. Smothers, C. Holly, C. Anderson, and J. Linville. 2006. Relative importance of wetland type versus anthropogenic activities in determining site invasibility. *Biological Invasions* 8: 1425–1432. doi:10.1007/s10530-006-0006-5.

Everitt, J. H., and C. Yang. 2007. Using QuickBird satellite imagery to distinguish two aquatic weeds in south Texas. *Journal of Aquatic Plant Management* 45: 25–31.

Ferrareze, M., L. Casatti, and M. G. Nogueira. 2014. Spatial heterogeneity affecting fish fauna in cascade reservoirs of the Upper Paraná Basin, Brazil. *Hydrobiologia* 738: 97–109. doi:10.1007/s10750-014-1922-5.

Firehun, Y., P. C. Struik, E. A. Lantinga, and T. Taye. 2013. Joint use of insects and fungal pathogens in the management of waterhyacinth (Eichhornia crassipes): Perspectives for Ethiopia. *Journal of Aquatic Plant Management*: 109–121.

Fraser, G. C. G., M. P. Hill, and J. A. Martin. 2016. Economic evaluation of water loss saving due to the biological control of water hyacinth at New Year's Dam, Eastern Cape province, South Africa. *African Journal of Aquatic Science* 41: 227–234. doi:10.2989/16085914.2016.1151765.

Giraldo, E., and A. Garzón. 2002. The potential for water hyacinth to improve the quality of Bogota River water in the Muña Reservoir: Comparison with the performance of waste stabilization ponds. *Water Science and Technology* 45: 103–110.

Gopal, B. 1987. *Water Hyacinth*. Amsterdam: Elsevier B.V.

Gopal, B., K. Sharma, and R. Trivedy. 1978. Studies on ecology and production in Indian freshwater ecosystems at primary producer level with emphasis on macrophytes. In *Glimpses of Ecology*, ed. J. Singh and B. Gopal, 349–376. Jaipur: International Science Publications.

Goshu, G., and S. Aynalem. 2017. Problem Overview of the Lake Tana Basin. In *Social and Ecological System Dynamics: Characteristics, Trends, and Integration in the Lake Tane Basin, Ethiopia*, ed. S. Stave, K and Goshu, G and Aynalem, 9–23. AESS Interdisciplinary Environmental Studies and Sciences Series. doi:10.1007/978-3-319-45755-0_2.

Gunaratne, A. M., S. Jayakody, and C. N. B. Bambaradeniya. 2009. Spatial distribution of aquatic birds in Anavilundawa Ramsar wetland sanctuary in Sri Lanka. *Biological Invasions* 11: 951–958. doi:10.1007/s10530-008-9307-1.

Gunaratne, A. M., S. Jayakody, and U. S. Amarasinghe. 2015. ``'Ornithological eutrophication'' as a source of allochthonous nutrient enrichment in Anavilundawa reservoir, Sri Lanka. *International Review of Hydrobiology* 100: 151–157. doi:10.1002/iroh.201501804.

Gunnarsson, C., and C. Mattsson. 1997. *Water hyacinth – trying to turn an environmental problem into an agricultural resource*. *Minor Field Studies-Swedish University of Agricultural Sciences, International Office (Sweden)*. Uppsala.

Hestir, E. L., S. Khanna, M. E. Andrew, M. J. Santos, J. H. Viers, J. A. Greenberg, S. S. Rajapakse, and S. L. Ustin. 2008. Identification of invasive vegetation using hyperspectral remote sensing in the California Delta ecosystem. *Remote Sensing of Environment* 112: 4034–4047. doi:10.1016/j.rse.2008.01.022.

Hill, M. P., and T. Olckers. 2000. Biological Control Initiatives against Water Hyacinth in South Africa: Constraining Factors, Success and New Courses of Action. *ACIAR proceedings* 102: 33–38.

Hopper, J. V., P. D. Pratt, K. F. McCue, M. J. Pitcairn, P. J. Moran, and J. D. Madsen. 2017. Spatial and temporal variation of biological control agents associated with Eichhornia crassipes in the Sacramento-San Joaquin River Delta, California. *Biological Control* 111. Elsevier: 13–22. doi:10.1016/j.biocontrol.2017.05.005.

Howard-Williams, C., and W. J. Junk. 1976. The decomposition of aquatic macrophytes in the floating meadows of a central Amazonian várzea lake. *Biogeographica* 7: 115–123.

Ingwani, E., T. Gumbo, and T. Gondo. 2010. The general information about the impact of water hyacinth on Aba Samuel dam, Addis Ababa, Ethiopia: Implications for ecohydrologists. *Ecohydrology and Hydrobiology* 10. Elsevier: 341–345. doi:10.2478/v10104-011-0014-7.

Jennerjahn, T. C. 2013. Does High Silicate Supply Control Phytoplankton Composition and Particulate Organic Matter Formation in Two Eutrophic Reservoirs in the Brantas River Catchment, Java, Indonesia? *Asian Journal of Water Environment and Pollution* 10: 41–53.

Jongmeesuk, A., V. Sanguanchaipaiwong, and D. Ochaikul. 2014. Pretreatment and Enzymatic Hydrolysis from Water Hyacinth (Eichhornia crassipes). *KMITL Science and Technology Journal* 14: 79–86.

Kassaye, Y. A., L. Skipperud, J. Einset, and B. Salbu. 2016. Aquatic macrophytes in Ethiopian Rift

Valley lakes; Their trace elements concentration and use as pollution indicators. *Aquatic Botany* 134: 18–25. doi:10.1016/j.aquabot.2016.06.004.

Katembo, N., M. P. Hill, and M. J. Byrne. 2013. Impacts of a sub-lethal dose of glyphosate on water hyacinth nutrients and its indirect effects on Neochetina weevils. *Biocontrol Science and Technology* 23: 1412–1426. doi:10.1080/09583157.2013.839982.

Khanna, S., M. J. Santos, E. L. Hestir, and S. L. Ustin. 2012. Plant community dynamics relative to the changing distribution of a highly invasive species, Eichhornia crassipes: A remote sensing perspective. *Biological Invasions* 14: 717–733. doi:10.1007/s10530-011-0112-x.

Knipling, E., S. West, and W. Haller. 1970. Growth characteristics, yield potential, and nutritive content of water hyacinths. *Soil Crop Sci Soc Fla Proc* 30: 51–63.

Kouamé, M. K., M. Y. Diétoa, S. K. Da Costa, E. O. Edia, A. Ouattara, and G. Gourene. 2010. Aquatic macroinvertebrate assemblages associated with root masses of water hyacinths, Eichhornia crassipes (Mart.) Solms-Laubach, 1883 (Commelinales: Pontederiaceae) in Taabo Lake, Ivory Coast. *Journal of Natural History* 44: 257–278. doi:10.1080/00222930903457208.

Kumar, J. I. N., H. Soni, and R. N. Kumar. 2008. Evaluation of biomonitoring approach to study lake contamination by accumulation of trace elements in selected aquatic macrophytes: A case study of Kanewal Community Reserve, Gujarat, India. *Applied Ecology and Environmental Research* 6: 65–76.

Lallana, V. H. 1980. Productividad de Eichhornia crassipes (Mart.) Solms. en una Laguna Isleña de la Cuenca del Río Paraná Medio. II. Biomasa y dinámica de población. *Ecología* 5: 1–16.

Lallana, V. H. 1981. Productividad de eichhornia crassipes. *Boletin de la Sociedad argentina de Botanica* 20: 99–107.

Lars, W. J., A. Maggie, and M. A. Christman. 2017. Invasive Aquatic Vegetation Management in the Sacramento – San Joaquin River Delta : Status and Recommendations. *San Francisco Estuary and Watershed Science* 15.

Leiming, C. 2006. Impact of floating vegetation in Shuikou impoundment, Minjian River, Fujian Province. *Journal of Lake Sciences* 18: 250–254.

Lopez, E. G. 1993. Effect of Glyphosate on different densities of water hyacinth. *Journal of Aquatic Plant Management* 31: 255–257.

Lu, J., J. Wu, Z. Fu, and L. Zhu. 2007. Water hyacinth in China: A sustainability science-based management framework. *Environmental Management* 40: 823–830. doi:10.1007/s00267-007-9003-4.

Magadza, C. H. D. 2003. Lake Chivero: A management case study. *Lakes and Reservoirs: Research and Management* 8: 69–81. doi:10.1046/j.1320-5331.2003.00214.x.

Mangas-Ramírez, E., and M. Elías-Gutiérrez. 2004. Effect of mechanical removal of water hyacinth (Eichhornia crassipes) on the water quality and biological communities in a Mexican reservoir. *Aquatic Ecosystem Health and Management* 7: 161–168. doi:10.1080/14634980490281597.

Marshall, B. E. 2005. The impact of eutrophication on Lake Chivero, Zimbabwe: A tropical African reservoir. In *Restoration and Management of Tropical Eutrophic Lakes*, ed. M. Reddy, 165–186. Boca Raton: CRC Press.

Martinez Jimenez, M., and M. A. Gomez Balandra. 2007. Integrated control of Eichhornia crassipes by using insects and plant pathogens in Mexico. *Crop Protection* 26: 1234–1238. doi:10.1016/j.cropro.2006.10.028.

Martins, D., R. A. Pitelli, M. S. Tomazella, R. H. Tanaka, and A. C. P. Rodrigues. 2009. Aquatic Plant Infestation Assessment in Porto Primavera Reservoir Before Final Filling. *Planta Daninha* 27:

879–886. doi:10.1590/S0100-83582009000500001.

Martins, D., E. D. Velini, N. Costa V, L. A. Cardoso, and G. S. F. Souza. 2011. Chemical Control of Eichhornia crassipes and Brachiaria subquadripara with Diquat under Reservoir Conditions. *Planta Daninha* 29: 51–57. doi:10.1590/S0100-83582011000100006.

Mishra, V. K., A. R. Upadhyay, S. K. Pandey, and B. D. Tripathi. 2008. Concentrations of heavy metals and aquatic macrophytes of Govind Ballabh Pant Sagar an anthropogenic lake affected by coal mining effluent. *Environmental monitoring and assessment* 141: 49–58. doi:10.1007/s10661-007-9877-x.

Moura Junior, E. G., A. Pott, W. Severi, and C. S. Zickel. 2019. Response of aquatic macrophyte biomass to limnological changes under water level fluctuation in tropical reservoirs. *Brazilian Journal of Biology* 79: 120–126. doi:10.1590/1519-6984.179656.

Muchini, R., W. Gumindoga, S. Togarepi, T. P. Masarira, and T. Dube. 2018. Near real time water quality monitoring of Chivero and Manyame lakes of Zimbabwe. *Proceedings of the International Association of Hydrological Sciences* 378: 85–92. doi:10.5194/piahs-378-85-2018.

Musil, C. F., and C. M. Breen. 1977. The influence of site and position in the plant community on the nutrient. *Hydrobiologia* 53: 67–72.

Neiff, A. P. de, J. Neiff, and A. Bonetto. 1977. Enemigos naturales de Eichhornia crassipes en el nordeste argentino y posibilidades de su aplicación al control biológico. *Ecosur* 4: 137–156.

Nguyen, T. H. T., P. Boets, K. Lock, M. N. D. Ambarita, M. A. E. Forio, P. Sasha, L. E. Dominguez-Granda, T. H. T. Hoang, et al. 2015. Habitat suitability of the invasive water hyacinth and its relation to water quality and macroinvertebrate diversity in a tropical reservoir. *Limnologica* 52: 67–74. doi:10.1016/j.limno.2015.03.006.

Ogutu-Ohwayo, R., K. Odongkara, W. Okello, D. Mbabazi, S. B. Wandera, L. M. Ndawula, and V. Natugonza. 2013. Variations and changes in habitat, productivity, composition of aquatic biota and fisheries of the Kyoga lake system: lessons for management. *African Journal of Aquatic Science* 38: 1–14. doi:10.2989/16085914.2013.795886.

Olivares, E., and G. Colonnello. 2000. Salinity gradient in the Manamo River, a dammed distributary of the Orinoco Delta, and its influence on the presence of Eichhornia crassipes and Paspalum repens. *Interciencia* 25: 242–248.

Oliveira, S. 1977. Produtividade primaria e evapotranspiracao da baronesa (Eichhornia crassipes) e alface d'agua (Pistia stratiotes) em condicoes de clima tropical [Bahia. Univ. Federal da Bahia.

Parra, J. V., and C. C. Hortenstine. 1974. Plant Nutritional Content of Some Florida Waterhyacints And Response By Pearl Millet to Incorporation of Waterhyacinths in three soil types. *Hyacinth control J* 12: 85–90.

Peixoto, R. B., H. Marotta, D. Bastviken, and A. Enrich-Prast. 2016. Floating Aquatic Macrophytes Can Substantially Offset Open Water CO2 Emissions from Tropical Floodplain Lake Ecosystems. *Ecosystems* 19: 724–736. doi:10.1007/s10021-016-9964-3.

Penfound, W. T. 1956. Primary Production of Vascular Aquatic Plants. *Limnology and Oceanography* 1: 92–101. doi:10.4319/lo.1956.1.2.0092.

Penfound, W. T., and T. T. Earle. 1948. The biology of the water hyacinth. *Ecological Monographs* 18: 447–472.

Perez del Viso, R., N. M. Tur, and V. Mantovani. 1968. Estimación de la biomasa de hidrófitos en cuencas isleñas del Paraná. *Physis* 28: 219–226.

Pinto-Coelho, R. M., and M. K. B. Greco. 1999. The contribution of water hyacinth (Eichhornia crassipes) and zooplankton to the internal cycling of phosphorus in the eutrophic Pampulha

Reservoir, Brazil. *Hydrobiologia* 411: 115–127. doi:10.1023/A:1003845516746.

Pitelli, R. L. C. M., A. M. C. M. Pitelli-Merenda, R. A. Pitelli, R. C. Siqueira, H. O. Barbosa, and L. Jesus. 2014a. Aquatic Macrophytes Community and Colonization on Aimores Reservoir. *Planta Daninha* 32: 475–482. doi:10.1590/S0100-83582014000300002.

Pitelli, R. L. C. M., R. A. Pitelli, C. J. Rodrigues, and J. H. P. Dias. 2014b. Aquatic plant community in Porto Primavera Reservoir. *Planta Daninha* 32: 467–473. doi:10.1590/S0100-83582014000300001.

Poddar, K., L. Mandal, and G. C. Banerjee. 1991. Studies on water hyacinth (Eichhornia crassipes) - chemical composition of the plant and water from different habitats. *Indian Veterinary Journal* 68: 833–837.

Polprasert, C., S. Wangsuphachart, and S. Muttamara. 1980. Composting nightsoil and water hyacinth in the tropics. *Compost science-land utilization* 21: 25–27.

Quintao, J. M. B., R. S. Rezende, and J. F. Goncalves Junior. 2013. Microbial effects in leaf breakdown in tropical reservoirs of different trophic status. *Freshwater Science* 32: 933–950. doi:10.1899/12-112.1.

Rahim, A., and T. R. Soeprobowati. 2019. Water Pollution Index of Batujai Reservoir, Central Lombok Regency-Indonesia. *Journal of Ecological Engineering* 20: 219–225. doi:10.12911/22998993/99822.

Rai, P. K. 2009. Heavy metals in water, sediments and wetland plants in an aquatic ecosystem of tropical industrial region, India. *ENVIRONMENTAL MONITORING AND ASSESSMENT* 158: 433–457. doi:10.1007/s10661-008-0595-9.

Reddy, K., and J. Tucker. 1983. Productivity and nutrient uptake of water hyacinth, Eichhornia crassipes I. Effect of nitrogen source. *Economic Botany* 37: 237–247. doi:10.1007/BF02858790.

Regina, N. M. M. 2012. Biological monitoring and pollution assessment of the Mukuvisi River, Harare, Zimbabwe. *Lakes and Reservoirs: Research and Management* 17: 73–80. doi:10.1111/j.1440-1770.2012.00497.x.

Rocha, F. C., E. M. Andrade, and F. B. Lopes. 2015. Water quality index calculated from biological, physical and chemical attributes. *Environmental monitoring and assessment* 187. doi:10.1007/s10661-014-4163-1.

Rodríguez-espinosa, P. F., V. C. Shruti, M. P. Jonathan, and E. Martinez-tavera. 2018. Ecotoxicology and Environmental Safety Metal concentrations and their potential ecological risks in fl uvial sediments of Atoyac River basin , Central Mexico : Volcanic and anthropogenic in fl uences. *Ecotoxicology and Environmental Safety* 148. Elsevier Inc.: 1020–1033. doi:10.1016/j.ecoenv.2017.11.068.

Rodríguez-Espinosa, P. F., J. A. Mendoza-Pérez, J. Tabla-Hernandez, E. Martínez-Tavera, and M. M. Monroy-Mendieta. 2018. Biodegradation and kinetics of organic compounds and heavy metals in an artificial wetland system (AWS) by using water hyacinths as a biological filter. *International Journal of Phytoremediation* 20: 35–43. doi:10.1080/15226514.2017.1328397.

Rodriguez, A., P. Avila-Perez, and I. D. Barcelo-Quintal. 1998. Bioaccumulation of chemical elements by water hyacinth (Eichhornia crassipes) found in Jose Antonio Alzate dam samples in the State of Mexico, Mexico. *Journal of Radioanalytical and Nuclear Chemistry* 238: 91–95. doi:10.1007/BF02385360.

Rodriguez, M., J. Brisson, G. Rueda, and M. S. Rodriguez. 2012. Water Quality Improvement of a Reservoir Invaded by an Exotic Macrophyte. *Invasive Plant Science and Management* 5: 290–299. doi:10.1614/IPSM-D-11-00023.1.

Rommens, W., J. Maes, N. Dekeza, P. Inghelbrecht, T. Nhiwatiwa, E. Holsters, F. Ollevier, B.

Marshall, et al. 2003. The impact of water hyacinth (Eichhornia crassipes) in a eutrophic subtropical impoundment (Lake Chivero, Zimbabwe). I. Water quality. *Archiv für Hydrobiologie* 158: 373–388. doi:10.1127/0003-9136/2003/0158-0373.

Sabattini, R. A., V. H. Lallana, and M. C. Marta. 1983. Inventario y biomasa de plantas acuáticas en un tramo del valle aluvial del río Paraná Medio. *Revista de la Asociación de Ciencias Naturales del Litoral* 14: 179–191.

Sahai, R., and A. B. Sinha. 1970. Contribution to the ecology of indian aquatics. *Hydrobiologia* 35: 376–382.

Santos, J. D. P., K. Galvis, L. D. Becerra, and C. A. Grattz. 2012. Problemas Ambientales en el Embalse de Hidroprado Tolima, Colombia. *Ingenio libre. Revista de la facultad de la Universidad Libre*: 79–81.

Santos, M. J., J. A. Greenberg, S. S. Rajapakse, M. E. Andrew, S. Khanna, L. W. J. Anderson, E. L. Hestir, and S. L. Ustin. 2009. Use of Hyperspectral Remote Sensing to Evaluate Efficacy of Aquatic Plant Management. *Invasive Plant Science and Management* 2: 216–229. doi:10.1614/ipsm-08-115.1.

Singh, S., and R. Sahai. 1979. Seasonal changes in the biomass of Eichhornia crassipes (Mart) solms in'Jalwania'pond of Gorakhpur [India]. *Indian Journal of Ecology* 6: 30–34.

Sinkala, T., E. T. Mwase, and M. Mwala. 2002. Control of aquatic weeds through pollutant reduction and weed utilization: A weed management approach in the lower Kafue River of Zambia. *Physics and Chemistry of the Earth* 27: 983–991. doi:10.1016/S1474-7065(02)00102-X.

Sunwoo, I. Y., J. E. Kwon, T. H. Nguyen, G. T. Jeong, and S. K. Kim. 2019. Ethanol production from water hyacinth (Eichhornia crassipes) hydrolysate by hyper-thermal acid hydrolysis, enzymatic saccharification and yeasts adapted to high concentration of xylose. *Bioprocess and Biosystems Engineering*. Springer Berlin Heidelberg. doi:10.1007/s00449-019-02136-3.

Téllez, T. R., E. M. de R. López, G. L. Granado, E. A. Pérez, R. M. López, and J. M. S. Guzmán. 2008. The water hyacinth, Eichhornia crassipes: An invasive plant in the Guadiana River Basin (Spain). *Aquatic Invasions* 3: 42–53. doi:10.3391/ai.2008.3.1.8.

Thamaga, K. H., and T. Dube. 2018. Testing two methods for mapping water hyacinth (Eichhornia crassipes) in the Greater Letaba river system, South Africa: discrimination and mapping potential of the polar-orbiting Sentinel-2 MSI and Landsat 8 OLI sensors. *International Journal of Remote Sensing* 39: 8041–8059. doi:10.1080/01431161.2018.1479796.

Thamasara, S. 1989. Problems and control of aquatic weeds in the irrigation systems of Thailand. *Journal of Aquatic Plant Management*.

Thi, T., B. Huyen, N. Ngoc, M. Trinh, N. Thi, and K. Cuc. Biodegradation Eichhornia crassipes of black soldier fly larvae (Hermetia illucens) based on growth rate and food consumption. *KHOA HỌC & CÔNG NGHỆ* 139: 131–136.

Thomaz, S. M., T. A. Pagioro, L. M. Bini, and K. J. Murphy. 2006. Effect of reservoir drawdown on biomass of three species of aquatic macrophytes in a large sub-tropical reservoir (Itaipu, Brazil). *Hydrobiologia* 570: 53–59. doi:10.1007/s10750-006-0161-9.

UNEP. 2013. *Water hyacinth – Can its aggressive invasion be controlled?* Vol. 7. Nairobi, Kenya: UNEP Global Environmental Alert Service.

Venugopal, G. 2002. Monitoring the Effects of Biological Control of Water Hyacinths Using Remotely Sensed Data: A Case Study of Bangalore, India. *Singapore Journal of Tropical Geography* 19: 91–105. doi:10.1111/1467-9493.00027.

Vilarrubia, T. V, and M. Cova. 1993. Study and ecological distribution od macrophytes in the Guri Reservoir, Venezuela. *Interciencia* 18: 77–82.

Villamagna, A. M., and B. R. Murphy. 2010. Ecological and socio-economic impacts of invasive water hyacinth (Eichhornia crassipes): A review. *Freshwater Biology* 55: 282–298. doi:10.1111/j.1365-2427.2009.02294.x.

Viriyavisuthisakul, S., P. Sanguansat, and T. Yamasaki. 2018. *Water Hyacinth Segmentation for Aquatic Weed Elimination in Thailand*. Edited by R DiBaja, GS and Gallo, L and Yetongnon, K and Dipanda, A and CastrillonSantana, M and Chbeir. *14th International Conference on Signal Image Technology & Internet based Systems (SITIS)*. Las Palmas: IEEE Comp Soc. doi:10.1109/SITIS.2018.00046.

Wang, Z., Z. Zhang, J. Zhang, Y. Zhang, H. Liu, and S. Yan. 2012. Large-scale utilization of water hyacinth for nutrient removal in Lake Dianchi in China: The effects on the water quality, macrozoobenthos and zooplankton. *Chemosphere* 89. Elsevier Ltd: 1255–1261. doi:10.1016/j.chemosphere.2012.08.001.

Wei, B., X. Yu, S. Zhang, and L. Gu. 2011. Comparison of the community structures of ammonia-oxidizing bacteria and archaea in rhizoplanes of floating aquatic macrophytes. *Microbiological Research* 166: 468–474. doi:10.1016/j.micres.2010.09.001.

Wersal, R. M., J. D. Madsen, and M. L. Tagert. 2006. Survey of Invasive and Native Aquatic Plants in the Ross Barnett Reservoir Survey of Invasive and Native Aquatic Plants in the Ross Barnett Reservoir. *Proceedings of the 36th Annual Mississippi Water Resources Conference*: 55–58.

Williams, A. E., and R. E. Hecky. 2005. Invasive aquatic weeds and eutrophication: The case of water hyacinth in Lake Victoria. In *Restoration and Management of Tropical Eutrophic Lakes*, ed. Reddy, MV, 187–225.

Wolverton, B. C., and R. C. Mcdonald. 1978. Nutritional composition of water hyacinths grown on domestic sewage. *Economic Botany* 32: 363–370. doi:10.1007/BF02907930.

Wooten, J. W., and J. D. Dodd. 1976. Growth of water hyacinths in treated sewage effluent. *Economic Botany* 30: 29–37.

Wulandari, D. A., D. Kurniani, S. Edhisono, F. Ardianto, and D. Dahlan. 2019. The effect of small dams in Rawa Pening catchment area on sedimentation rate of Rawa Pening Lake. In *2nd Conference for Civil Engineering Research Networks (CONCERN-2 2018)*, ed. P. Wirahadikusumah, RD and Hasiholan, B and Kusumaningrum. Vol. 270. MATEC Web of Conferences. doi:10.1051/matecconf/201927004018.

WWF. 2017. *Kafue Flats Status Report. Monitoring. Monitoring the pulse of the blue heart of Zambia's economy*. Lusaka, Zambia: WWF Zambia.

Xiao, C., P. Wenbin, and W. Mu. 2002. Spatial distribution characteristics and dynamics of Eichhornia crassipes in the Shuikou Reservoir, Fujian Province. *Journal of Lake Sciences* 24: 391–399.

Xie, Y., H. Qin, and D. Yu. 2004. Nutrient limitation to the decomposition of water hyacinth (Eichhornia crassipes). *Hydrobiologia* 529: 105–112. doi:10.1007/s10750-004-5494-7.

Xiong, W., H. Wang, Q. Wang, J. Tang, P. A. Bowler, D. Xie, L. Pan, and Z. Wang. 2018. Non-native species in the Three Gorges Dam Reservoir : status and risks. *BioInvasions Records* 7: 153–158.

Zhang, Y., H. Liu, S. Yan, X. Wen, H. Qin, Z. Wang, and Z. Zhang. 2019. Phosphorus removal from the hyper-eutrophic lake caohai (China) with large-scale water hyacinth cultivation. *Environmental Science and Pollution Research* 26: 12975–12984. doi:10.1007/s11356-019-04469-8.
